# Expression and Signaling Pathways of Nerve Growth Factor (NGF) and Pro-NGF in Breast Cancer: A Systematic Review

**DOI:** 10.3390/curroncol29110640

**Published:** 2022-10-27

**Authors:** Francesco Bruno, Domenico Arcuri, Francesca Vozzo, Antonio Malvaso, Alberto Montesanto, Raffaele Maletta

**Affiliations:** 1 Department of Primary Care, Regional Neurogenetic Centre (CRN), ASP Catanzaro, 88046 Lamezia Terme, Italy; 2 Association for Neurogenetic Research (ARN), 88046 Lamezia Terme, Italy; 3 Department of Medical and Surgical Sciences, Magna Graecia University of Catanzaro, 88050 Catanzaro, Italy; 4 Faculty of Medicine and Surgery, Vita-Salute San Raffaele University, 20132 Milan, Italy; 5 Department of Biology, Ecology and Earth Sciences, University of Calabria, 87036 Rende, Italy

**Keywords:** breast cancer, nerve growth factor (NGF), TrkA, p75NTR, NGFR, pro-NGF, angiogenesis, invasion, metastasis, diagnosis, prognosis, treatment

## Abstract

Breast cancer represents the most common type of cancer and is the leading cause of death due to cancer among women. Thus, the prevention and early diagnosis of breast cancer is of primary urgency, as well as the development of new treatments able to improve its prognosis. Nerve Growth Factor (NGF) is a neurotrophic factor involved in the regulation of neuronal functions through the binding of the Tropomyosin receptor kinase A (TrkA) and the Nerve Growth Factor receptor or Pan-Neurotrophin Receptor 75 (NGFR/p75NTR). In addition, its precursor (pro-NGF) can extert biological activity by forming a trimeric complex with NGFR/p75NTR and sortilin, or by binding to TrkA receptors with low affinity. Several examples of in vitro and in vivo evidence show that NGF is both synthesized and released by breast cancer cells, and has mitogen, antiapoptotic and angiogenic effects on these cells through the activation of different signaling cascades that involve TrkA and NGFR/p75NTR receptors. Conversely, pro-NGF signaling has been related to breast cancer invasion and metastasis. Other studies suggested that NGF and its receptors could represent a good diagnostic and prognostic tool, as well as promising therapeutic targets for breast cancer. In this paper, we comprehensively summarize and systematically review the current experimental evidence on this topic. INPLASY ID: INPLASY2022100017.

## 1. Introduction

Breast cancer is a neoplasm of epithelial origin that generally develops in the parts of the breast tissue made up of the glands involved in milk production, or in the ducts that connect the glands to the nipple. In women, it represents the most frequent cancer [1], with 2.3 million of new cases diagnosed worldwide in 2020 [2], as well as the leading cause of cancer death [3]. It is estimated that the incidence of breast cancer will increase over the years, and will reach 3.2 million in 2050 [4], thus representing a health emergency both from a medical [5] and a psychological point of view [6,7]. Therefore, prevention and early diagnosis of breast cancer is of primary urgency, as well as the development of new treatments able to improve its prognosis.

Nerve Growth Factor (NGF) is a neurotrophic factor involved in the regulation of neuronal functions thought the binding to the Tropomyosin receptor kinase A (TrkA) and to the Nerve Growth Factor Receptor or Pan-Neurotrophin Receptor 75 (NGFR/p75NTR) [8]. Since the 1990s, several studies have indicated that NGF and its receptors could also play a key role in the pathogenesis of breast cancer and, consequently, could represent a new therapeutic target [9,10,11,12,13]. Other evidence indicates that both NGF and its receptors could be considered accurate diagnostic and prognostic tools for breast cancer [14,15,16,17]. Moreover, the NGF precursor (pro-NGF) signaling pathways were related to breast cancer invasion and metastasis [18,19].

However, as far as we know, this topic has never been systematically reviewed. After providing a general overview of breast cancer and NGF signaling pathways, here we systematically review and comprehensively summarize the current experimental evidence regarding the involvement of NGF signaling pathways in breast cancer. Based on the findings, significant issues for future studies are then put forward.

## 2. Breast Cancer: An Overview

### 2.1. Classification

There are several classifications of breast cancers. The main classifications are based on: (i) histologic type, (ii) grading; (iii) immunophenotype; (iv) tumor size (T), nodal status (N), and distant metastasis (M) staging (TNM classification); and (v) molecular subtype [20]. (Table 1).

#### 2.1.1. Histological Type

In accordance with the histological classification, breast cancer can be subdivided into carcinoma in situ and invasive or infiltrating carcinoma [21]. Carcinomas in situ are further sub-classified into ductal (DCIS) and lobular (CLIS). In turn, DCSI is further sub-categorized into: comedo, cribiform, micropalillary, papillary, and solid [22], whereas CLIS is sub-categorized into: tubular, ductal lobular, invasive lobular, infiltrating ductal (e.g., intracanalar, apocrine), mucinous (colloid), and medullary [21,23,24].

#### 2.1.2. Grading

The histological grade—also known as grading—defines whether a neoplasm is well, moderately, or poorly differentiated, and therefore constitutes a fundamental parameter within which to evaluate each newly diagnosed breast cancer [25,26]. The extent of differentiation is assessed by pathologists through the observation of several morphological characteristics, such as: the degree of formation of tubular structures, nuclear pleomorphism, and proliferation, as indicated by mitotic index [20,27]. The most widely used grading system worldwide is the Nottingham system (or Scarff-Bloom-Richardson method, modified by Elston-Ellis) which evaluates all three parameters previously mentioned. A score ranging from 1 to 3 is assigned to each of these parameters; the sum of the three scores determines a global score, based on which the histological grade is defined: (i) grade 1 (G1), well differentiated tumor (score from 3 to 5); (ii) grade 2 (G2), moderately differentiated tumor (score 6 or 7); (iii) grade 3 (G3), poorly differentiated tumor (score 8 or 9) [25].

#### 2.1.3. Immunophenotype

Regarding the immunophenotype classification, the presence/absence of molecular markers for estrogen receptors (ER), progesterone receptors (PR) (i.e., hormone receptors, HR), and human epidermal growth factor 2 (HER2), breast cancer can also be classified into: (i) HR positive/HER2 negative (about 70% of cases); (ii) HER2 positive (about 15–20% of cases) and; (iii) triple-negative (tumors lacking all three standard molecular markers, about 15% of the remaining cases) [28].

#### 2.1.4. TNM Classification

The TNM classification was proposed by the American Joint Committee on Cancer and is essentially based on three variables: tumor size (T), lymph node involvement (N) and presence of metastases (M) [21,29]. The last version also incorporates into the staging system several biological factors such as ER and PR receptors, HER2, histological grade and multigene prognostic assays. Combining these factors, the breast cancer can be classified into one of five stages indicated with the Roman numerals I, II, III, and IV (plus 0) [20,29].

#### 2.1.5. Molecular Subtype

From a molecular point of view, a distinction can be made among several sub-categories of breast cancer, identified through global gene expression profiling studies: luminal A, luminal B, basal, claudin-low and HER2-enriched [20,21].

The names for luminal A and B derive from the gene expression profile which share some similarities with the normal luminal epithelium of the breast. However, high expression levels of ER-related genes are observed in luminal A tumors, as well as low expression of HER2 genes and low expression of proliferation-related genes. On the contrary, a low expression of ER-related genes, variable expression of HER2-related genes, and high expression of proliferation-related genes were observed in luminal B [30,31]. For this reason, the proportion of the proliferation genes/cells is a useful parameter for the differential diagnosis between luminal A and B tumors [32].

The basal subtype name is derived from the gene expression profile which share some similarities with the basal epithelial cells. However, a low expression of the luminal and HER2 gene cluster also characterized this specific subtype [33]. Moreover, while most triple-negative breast cancers are basal, not all basal-like forms are triple-negative; consequently, there is a considerable discordance between these two classification methods that must always be considered [34,35]. In addition to the basal breast cancer, the claudin-low are also primarily triple-negative, despite the fact that a limited number of triple-negative breast cancers are claudin-low [36]. In addition, a low expression of genes involved in cell—cell adhesion, such as claudin 3, claudin 4, claudin 7, occludin and E-cadherin, is usually observed in this specific sub-type [1]. Finally, high expression levels of the HER2 gene cluster and proliferation genes characterize the HER2-enriched subtype, which also showed a low level of genes, usually observed in the luminal and the basal subtype [37].

### 2.2. Risk Factors

The literature suggests that several risk factors are associated with breast cancer. These are commonly classified in non-modifiable and modifiable factors. Major non-modifiable factors include the female gender, older age, ethnicity (i.e., white non-Hispanic women are the most affected), a family or personal history of breast cancer, presence of genetic mutations (mostly in *BRCA1* and *BRCA2* genes), prior radiation therapy history, reproductive factors (e.g., pregnancy characteristics, late age of menopause) (for a review see: [1,38]). The main modifiable factors include lifestyle (e.g., obesity, diabetes, alcohol consumption and smoking), poorer vitamin supplementation, hormonal contraceptive or post-menopausal methods, air pollution and night work (for a review see: [1,38]).

### 2.3. Diagnosis

The standard diagnostic process of breast cancer includes anamnesis, clinical examinations, and medical imaging (e.g., mammography, ultrasonography, magnetic resonance imaging) [39]. Among the medical imaging techniques, mammography is commonly used as a screening tool in many countries, although several researchers are increasingly emphasizing its limitations, such as the radiation exposition, the low sensitivity in women with dense breast tissue [40], the high false-positive and false-negative rates [41], and the disadvantageous cost-effectiveness ratio [42]. In addition, it has been shown that molecular biotechnology examinations—aimed to detect specific biomarkers, such as nucleic acid, proteins, cells and tissues—can diagnose breast cancer earlier than imaging techniques, and therefore are becoming increasingly auxiliary methods used to diagnose breast cancer, and one of the most research topics in this field (for a review see: [39,41]).

### 2.4. Treatments and Prognosis

Given the heterogeneity of breast cancer, treatment strategies are chosen based on the type and the extent of the cancer. These include surgery [1], chemotherapy [28], radiotherapy [1], hormonal therapy (i.e., selective ER modulators, such as tamoxifen, toremifene; selective ER degraders such as fulvestrant; aromatase inhibitors such as letrozole, anastrazole, ex-emestane) [43,44] and biological therapy [45].

The advancement in knowledge on breast cancer and the use of personalized medicine have greatly improved the prognosis and survival rates compared to previous decades [46]. However, as already mentioned, breast cancer continues to be the main cause of cancer mortality in women, with an estimation of 685,000 deaths in 2020 alone [2], prompting researchers to conduct research on new treatments, especially for the advanced stages, presence of metastasis or cancer types with a poorer prognosis due to chemoresistance, such as triple-negative [47,48].

Classical prognostic factors of breast cancer include age, tumor stage, type and lymphovascular status [49]. In addition to these features, the American Joint Committee on Cancer (AJCC) also recommend the evaluation of some biological factors (i.e., ER, PR, HER2, grade, and multigene assays) to define the prognosis [50]. In addition, Ki67, a protein expressed only during cell proliferation, is frequently used to predict the prognosis [51]. Other novel and promising prognostic molecular markers are represented by p53, p14ARF, cyclin D1, cyclin E, *TBX2/3*, *BRCA1/2*, and *VEGF* (for a review see: [52]).

## 3. NGF Signaling Pathways

Neurotrophins are a set of proteins that play a pivotal role in the growth, survival, and differentiation of central and peripheral neurons [53,54,55,56], as well as in the apoptotic Programmed Cell Death (PCD) [57]. Four types of neurotrophins have been identified in mammals: NGF, Brain-Derived Neurotrophic Factor (BDNF), Neurotrophin 3 (NT-3) and Neurotrophin 4 (NT-4) [58]. NGF were isolated in the 1950s by Rita Levi Montalcini [59] and Stanley Cohen [60], a discovery for which they won the Nobel Prize for Medicine in 1986.

The human *NGF* gene is located on the short arm of chromosome 1 (1p22) and encodes for pro-NGF, a protein which has its own biological activities [61]. In turn, pro-NGF can be cleaved by proteases (e.g., plasmin, furin, matrix metalloproteinase MMP7 and MMP3) to produce the mature NGF form [62].

In addition to neurons, research has also focused on the role of NGF in non-neural functions, highlighting its presence and activity in the reproductive, endocrine, cardiovascular, and immune systems [10,63]. NGF is also synthesized, stored and released by vascular endothelial cells, platelets, fibroblasts [64,65,66,67] and cancer cells [10]. In particular, NGF exerts its function by binding two receptors: TrkA and NGFR/p75NTR.

In 1986, the TrkA receptor was isolated from a human colon carcinoma [68] and is encoded by the *NTRK1* gene [69,70], located on chromosome 1 (1q21-22) [61,71], in the same manner of the *NGF* gene. Several lines of evidence underline that the binding of NGF to TrkA receptor mediates proliferation, differentiation and survival of both neurons and cancer cells via the activation of PI3K/Akt, Ras/MAPK and PLCγ pathways [10].

The second receptor related to NGF is the tumor necrosis factor NGFR/p75NTR, first cloned in 1986 [72], and encoded from the *NGFR* gene located on chromosome 17 (17q21.33) [73]. The binding of NGF to NGFR/p75NTR leads to the activation of NF-kB or JNK, which mediates opposite effects on survival and apoptosis of both neurons and cancer cells, respectively [10]. The biological effects of NGFR/p75NTR are determined by the level of expression of TrkA receptors. Whether the TrkA receptors are not expressed or under-expressed, NGFR/p75NTR induces apoptotic signals. On the other hand, when NGFR/p75NTR and TrkA receptors are co-expressed, NGFR/p75NTR increases the affinity of the TrkA receptor for NGF, and thus mediate the activation of survival pathways [74,75,76,77].

As previously mentioned, pro-NGF also exhibit neurotrophic activity. In particular, pro-NGF can exert proapoptotic effects by forming a trimeric complex with its high affinity NGFR/p75NTR and sortilin receptors [78,79]. Interestingly, pro-NGF can also bind with low affinity to TrkA receptors, therefore inducing signaling of cells survival [80].

## 4. Methods

This systematic review was conducted following the Preferred Reporting Items for Systematic Review and Meta-Analysis (PRISMA) guidelines [81]. We registered the protocol on the International Platform of Registered Systematic Review and Meta-analysis Protocols (registration number: INPLASY2022100017).

### 4.1. Study Search

A systematic search was carried out in EMBASE, PUBMED and COCHRANE databases. A manual search in the bibliographies of selected articles was also conducted. The following Boolean search string was used, considering free text and Medical Subject Heading (MeSH) terms: (“breast cancer”) AND (“nerve growth factor” OR “NGF”) OR (“nerve growth factor precursor”) OR (“nerve growth factor receptor” OR “NGFR”) OR (“tropomyosin receptor kinase A” OR “TrkA”) OR (“sortilin”) synonyms. All returned results were systematically identified, screened, then extracted for relevant information following the PRISMA guidelines [81].

### 4.2. Exclusion and Inclusion Criteria

All studies with the aim of understanding the role of pro-NGF, NGF and its receptors in breast cancer were included in the systematic review. Articles that did not include original research (e.g., review, opinion article or conference abstract), articles with an unclear design of the study and studies that did not include the role of NGF in breast cancer were excluded from further analysis. Titles, abstracts, and articles were evaluated by two separate reviewers (A.Ma. and F.B.). Titles and abstract were reviewed for subject relevance. The investigators read full-text versions of eligible articles on their own. Disagreements were addressed by consensus between the two reviewers. A third investigator (R.M.) was consulted if the two reviewers reached different decisions or when in doubt. Additional research was obtained via appropriate publication reference lists and by consulting a specialist in the field.

## 5. Results

### 5.1. Included Studies

**Figure 1:**
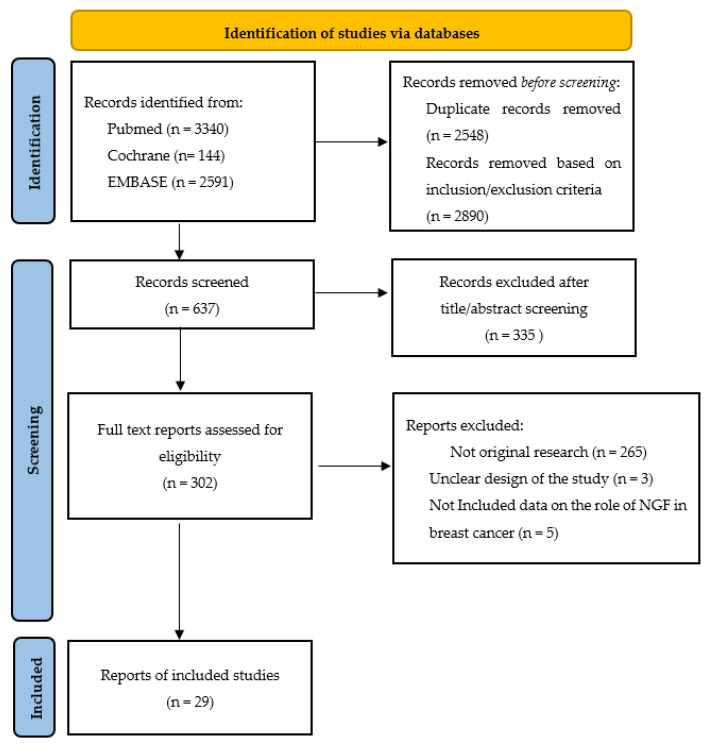
The flowchart of the studies selection.

The systematic search in the three databases generated 6075 entries. After removing duplicates (*n* = 2548), 3527 records were initially screened. After applying the inclusion and exclusion criteria, 2890 records were removed for a total of 637 remaining record to be screened. Of these, 335 records were excluded based on the title and/or abstract. We reviewed the full text of the remaining 302 articles with the subsequent removal of 265 articles that did not include original research (e.g., review, opinion article or conference abstract); three articles with unclear study design or setting of the study; and five articles that did not include specific data on the role of NGF in breast cancer (Figure 1 and Table 2).

### 5.2. NGF and Its Receptors Expression in Breast Cancer

Contrary to normal breast epithelial cells, both pro-NGF and NGF are synthesized and released by breast cancer cells [18,82,92]. In particular, Dollè et al. [92] found an overexpression of NGF either at the transcriptional or protein level, as well as the presence of NGF within classical secretion vesicles in several breast cancer cell lines and breast tumor biopsies of invasive ductal carcinoma. The authors also showed that NGF is biologically active and acts as an autocrine factor for breast cancer development. In 2008, Adriaenssens et al. [82] confirmed the high expression of NGF, particularly concentered in the epithelial cancer cells, in several histological types of breast cancer (i.e., invasive ductal, invasive lobular, colloid, apocrine, epidermoid metaplastic, tubular and intracanal carcinomas), through the immunostaining with anti-NGF of human breast cancer tissue sections obtained from a series of biopsies.

More recently, Kumar et al. [94] reported an overexpression of NGF in benign phyllodes, a rare fibro-epithelial breast tumor, demonstrating that this neurotrophin could play a role beyond the pathogenesis of malignant tumors. In addition to NGF, NGFR/p75NTR and TrkA receptors are also expressed in breast cancers cells, either at the transcriptional or protein level [83,84,89,95].

### 5.3. Mitogenesis of Breast Cancer Cells

In 1998, through the use of MCF-7 and MDAMB-231 cell lines for the study of invasive ductal and triple-negative breast cancers (TNBC), respectively, Descamps et al. [88] showed that NGF not only induced cells in the G0 phase to reenter the cell cycle, but also reduced the duration of the cell cycle. The NGF-stimulated proliferation seems to happen in a concentration-dependent manner, at least in MCF-7 cells [85]. Moreover, Sakamoto et al. [99] reported a correlation between NGF and cell proliferation assessed by Ki67 index, in 71 specimens of invasive ductal carcinoma.

These effects are most likely due to the activation of the TrkA/Ras/MAPK cascade [90], as well as the NGF-induced phosphorylation of P185^HER2^ [100], a kinase receptor *per se*, which are overexpressed in breast cancer cells and also stimulate their cell growth through the MAPK pathway [104,105,106]. Com et al. [86] also reported a list of TrkA signaling partners in breast cancer cells, such as IQGAP1m VCP and actin, by using proteomics on the MCF-7 cell line. The authors speculated that IQGAP1 could also represent a scaffold protein in the TrkA/MAPK mitogenic pathway and that VCP and actin proteins could be involved in the TrkA/PI3K/Akt pathway, although other studies are needed to confirm this hypothesis. In addition, it has been shown that breast cancer cells overexpressing TrkA show increased tumorgenicity [70] and that, in TNBC cells, NGF activated the TrkA receptors, leading to the formation of the TrkA/β1-integrin/FAK/Src complex involved in mitogenesis [91]. Moreover, Bashir et al. [84] found an upregulation of NGFR/p75NTR in breast cancer tissue, MCF7 cell line and isolated cancer stem cells (MCF7-CSCs) that induces the activation of NF-κB pathway in order to mediate cell proliferation.

### 5.4. Anti-Apoptosis and Survival of Breast Cancer Cells

As previously mentioned, the binding of NGF to NGFR/p75NTR can activate the NF-kB signaling pathway for the regulation of cell survival [10]. The same pathway could be involved in the anti-apoptosis effect of NGF on breast cancer cells, as demonstrated by Descamps et al. [90] and Bashir et al. [84]. These data are in line with the results of Naderi et al. [97] and Chakravarthy et al. [17] on a subset of ER-positive breast cancer with overexpression of *BEX2* gene and in TNBC cells line, respectively. Conversely, Sakamoto et al. [99] found a correlation between the apoptotic index and the expression of NGFR/p75NTR in 71 specimens of human invasive ductal breast carcinoma.

According to Com et al. [86], the TrkA receptor may also play a role in the NGF-induced anti-apoptotic cascade by involving the DNA repair protein Ku70. In particular, the authors showed that TrkA and Ku70 co-localize and interact upon NGF stimulation. This could lead to the TrkA-mediated tyrosine phosphorylation of Ku-70 and, thus, to MCF-7 breast cancer cells survival. Furthermore, Ku70 depletion induces a strong potentiation of apoptosis in TrkA-overexpressing cells. For this reason, Ku70 might be considered as a promising therapeutic target to induce the selective apoptosis of breast cancer cells overexpressing TrkA, although further in vitro and in vivo studies are required to confirm this hypothesis. Finally, Com et al. [86] showed that nucleophosmin, a protein involved in cell growth and proliferation, could be involved in the cytoprotective activities of NGF upon TrkA activation in breast cancer cells.

### 5.5. Angiogenesis of Breast Cancer

Tumor angiogenesis refers to the process characterized by a proliferation of new blood vessels from existing ones, to supply nutrients to the cancer [107]. The Vascular Endothelial Growth Factor (VEGF) is a potent angiogenic factor in breast cancer [108,109]. Interestingly, Romon et al. [98] showed that NGF stimulate the breast cancer angiogenesis by activating multiple TrkA signaling pathways, as well as promoting the secretion of VEGF in both endothelial and breast cancer cells. Moreover, Lagadec et al. [95] also showed that the increased tumor angiogenesis of breast cancer cells was related to TrkA overexpression.

### 5.6. Breast Cancer Invasion and Metastasis

Invasion and metastasis are extremely complex processes involving different classes of proteins, such as enzymes (extracellular proteases), glycoproteins (integrins), and immunoglobulins [110]. These processes cause biological changes in the functioning of the cells that begin from tumor invasion into blood and lymph vessels adjacent to the primary tumor site to metastatic colonization at other sites of the body [111].

Due to their proliferative and anti-apoptotic activities, NGF and its receptors seem to also be involved in the invasion and metastasis processes of breast cancer cells. The involvement of NGF receptors in breast cancer was first demonstrated by Aragona et al. [83], who observed a rapid metastatic spreading in NGFR/p75NTR-negative breast cancer patients. Some years later, Davidson et al. [87] characterized the expression of NGF, NGFR/p75NTR and phospho-TrkA (p-TrkA activated receptor) during the progression of breast carcinoma from primary tumor to pleural effusion in sections from malignant pleural effusions from breast cancer patients and the corresponding solid tumors. From the results of this study emerged the observation that NGF expression in effusions significantly predicted a shorter time to progression (TTP). In addition, the authors reported a downregulation and an upregulation of NGFR/p75NTR and TrkA, respectively, compared to primary breast tumors. The levels of TrkA were also upregulated in locoregional recurrences compared to early lymph node metastases [87]. The involvement of NGF in breast cancer was further demonstrated by Romon et al. [98], who observed that in endothelial cells, this neurotrophin is able to significantly influence some important processes involved in tumor angiogenesis that include invasion, cord formation and the monolayer permeability. In addition, a metastatic effect on xenografted breast cancer cells in immunodeficient mice was also reported [95]. In particular, Lagadec et al. [95] observed bigger metastatic foci in the lungs, liver and brain of mice that received TrkA overexpressing cells, most likely due to the overexpression of TrkA receptor, thus relating to the activation of the downstream PI3K/Akt and Ras/MAPK signaling pathways [70,95,98], as well as to the NGF-induced increased secretion of VEGF [98]. Trouvilliez et al. [101] demonstrated that NGF stimulation also causes the binding of the v3 isoform of CD44 to the TrkA receptors, leading to breast cancer cell tumor development and metastasis in vivo. Regarding triple-negative breast cancers, Di Donato et al. [91], by using MDA-MB-231 and MDA-MB-453 cells line, found that the NGF-induced activation of the TrkA receptor results in the formation of the TrkA/β1-integrin/FAK/Src complex, which leads to cell migration and invasion, and to increased spheroid size. In addition, a recent study in a population of TNBC cells reported an overexpression of NGFR/p75NTR and an interaction between NGFR/p75NTR and TrkC receptors which affects tumor growth and metastasis through the Trk MEK-ERK1-ZEB1 and PI3K-AKT signaling pathways [15]. Moreover, NGFR/p75NTR appears to also be involved in the proliferation and metastatization of invasive ductal carcinoma through the NF-kB pathway, as demonstrated on MCF-7 cell [84].

In addition to NGF, two studies [18,19] demonstrated the involvement of pro-NGF signaling pathways in breast cancer invasion and metastasis. Demont et al. [18] documented the overexpression of pro-NGF and its biding to TrkA plus sortilin in breast cancer cells. This autocrine loop leads to the activation of Akt and Src, and thus to the stimulation of the breast cancer cell lymph node invasion in breast cancer cells. Interestingly, the authors also reported that the pro-NGF appears to have a greater invasive effect than mature NGF [18]. Moreover, Lévêque et al. [19] showed that the binding of pro-NGF to sortilin leads to the formation of a sortilin/TrkA/EphA2 complex that induces cell invasion.

### 5.7. NGF and Its Receptors as Diagnostic Markers for Breast Cancer

As mentioned above, several evidence indicated that NGF is synthesized and released by breast cancer cells, but not from normal breast epithelial cells [18,82,92]. In particular, protein and immunological expression of NGF have been reported in the majority of human breast tumors [82], making it a broader diagnostic potential than ER or HER-2 [17]. Research has also shown that TrkA and NGFR/p75NTR receptors are overexpressed in most types of breast cancer compared to normal cells [87,89]. Moreover, Islam et al. [14] demonstrated that Russell’s Viper Venom (RVV)-NGFa (an NGF isoform), labelled with Fluorescein Isothiocyanate (FITC), establishes strong binding to TrkA and NGFR/p75NTR receptors in breast cancer cells but not in non-cancerous cells. This is a promising result for the future development of a tool using a fluorescent molecule-tagged RVV-NGFa binding technique to differentiate between cancerous and non-cancerous cells, and thus to diagnose breast cancer [14].

In addition to representing a potential new biomarker for the diagnosis of breast cancer, the analysis of NGF and its receptors could also be useful for making the differential diagnosis between various types of breast cancer. Tsang et al. [102] reported that NGFR/p75NTR could represent a potential marker for specific molecular subtypes of breast cancer through the comparison of its immunohistochemical expression in 602 specimens of luminal A, luminal B, HER2-overexpressed, basal-like and unclassified subtypes. From this study, it emerged that the NGFR/p75NTR expression was positively correlated with basal markers, including Ki67, Cytokeratin (CK5/6), CK14, p63, c-kit and EGFR, but negatively with HR. Regarding the molecular subtypes, NGF was positively associated with luminal B and basal-like breast cancer, with a comparable or better specificity than other basal markers or ER, PR, HER2 and Ki67, respectively. NGF was also negatively associated with luminal A. In addition, the results of Wu et al. [15] provide the basis for better characterization and use of NGFR/p75NTR as a diagnostic marker for determining the metastatic potential of TNBC cells in the future. Finally, the study of Kumar et al. [94] opens the prospect of using NGF as a biomarker to also distinguish benign tumors from each other, as breast phyllodes tumors overexpress NGF up to five times more than fibroadenomas tumors.

### 5.8. NGF and Its Receptors as Prognostic Markers for Breast Cancer

The prognostic value of NGF, TrkA and NGFR/p75NTR expression was evaluated in several studies [15,16,17,19,83,87,89,93,99].

Davidson et al. [87] posed NGF as the first molecular marker able to predict the time interval to progression. Namely, the authors found a mean of time to progression of 6.3 and of 4 years for NGF-negative and -positive effusions, respectively. Noh et al. [16] analyzed the immunohistochemical expression of NGF in breast cancer tissues obtained from 145 women affected by invasive ductal carcinomas (*n* = 137) and invasive lobular carcinomas (*n* = 8). The level of NGF found was significantly associated with heme oxygenase-1 (HO1) expression, histologic grade, HER2 expression, and latent distant metastasis. In addition, it predicted shorter overall survival and relapse-free survival. A recent study also provided the first evidence of the unfavorable prognostic value of the high expression of NGF in serum-derived exomes in a cohort of 129 patients mainly affected by invasive ductal carcinoma (96.9%) undergoing to neoadjuvant chemotherapy [93].

Regarding NGF receptors, Descamps et al. [89] reported that the overall TrkA mRNA expression predicts a more favorable prognosis in a highly variable cohort of 363 primary breast carcinoma. However, as mentioned above, Davidson et al. [87] showed that the levels of p-TrkA were associated with tumor progression to effusion in metastatic breast carcinoma, thus correlating the dysregulation of p-TrkA with poor prognostic outcome in a more uniform cohort of 39 patients. Furthermore, the immunohistochemical expression of NGFR/p75NTR receptor was reported as a positive and negative prognostic factor for non-TNBC and TNBC, respectively. By analyzing the tissues of 46 patients affected by different histological types of breast cancer (i.e., infiltrating ductal not otherwise specified, infiltrating ductal comedo, lobular invasive, mucinous, medullary), the expression of NGFR/p75NTR was associated with a longer disease-free survival, in addition to ER positivity, small tumor dimension, low histologic grade (G1–G2), old age and menopause [83]. In the same way, by analyzing 71 specimens of invasive ductal carcinoma, Sakamoto et al. [99] found that immunohistochemical NGF-positive and NGFR/p75NTR-negative show a lower disease-free survival rate, whereas the opposite pattern was associated with a more favorable outcome. Wu et al. [15] found an overexpression of NGFR/p75NTR in TNBC patients that negatively correlated with their overall survival. Indeed, it has been reported that the upregulation of NGFR/p75NTR mediated by NGF can contribute to chemoresistance [17]. As evidence of this finding, a sub-type of ER-positive breast cancer with an overexpression of the *BEX2* gene and treated with tamoxifen reported a more favorable prognosis as *BEX2* modulates the activation of NF-kB due to NGFR/p75NTR and enhances the antiproliferative effect of tamoxifen [97]. Lévêque et al. [19] demonstrated that high TrkA/EphA2 levels and a pro-NGF-induced complex were associated with poor prognosis in breast cancer patients. Finally, Jung et al. [93] also reported an alternation of DNA copy number amplifications and mRNA upregulation of NGF that was correlated with a worse survival in the same cohort of patients manly affected by invasive ductal carcinoma and who underwent neoadjuvant chemotherapy.

### 5.9. NGF Signaling Pathways as A Therapeutic Target for Breast Cancer

Preliminary data showed that NGF and its receptors could represent a promising target for the treatment of breast cancer. In particular, Adriaenssens et al. [82] reported that both anti-NGF antibodies and small interfering RNA (siRNA) against NGF reduce the cell proliferation, increase apoptosis and inhibit angiogenesis and metastasis of breast cancer. In addition, Dollè et al. [92] showed that the use of anti-NGF antibodies led to a dose-response reduction of the constitutive growth of breast cancer cell lines. As mentioned above, Romon et al. [98] reported that NGF increased the secretion of VEGF in endothelial and breast cancer cells. Interestingly, in vivo experiments showed that the administration of anti-VEGF antibodies significantly reduced the NGF-induced cell invasion and angiogenesis. In addition, the treatment of TNBC cells with anti-NGF antibodies or NGF inhibitors (i.e., Ro 08-2750 and Y1086) reduced the NGF-induced increased levels of NGFR/p75NTR, known to be involved in anti-apoptosis [17].

Through the use of several cell lines, it has been shown that NGF -mediated proliferation of breast cancer cells could be reduced or inhibited by the TrkA phosphorylation inhibitor K252a [85,88], the selective inhibitor of the MAPK cascade PD98059 [88], the antiestrogen drug tamoxifen [85], the TrkA inhibitor larotrectinib [70] and endocannabinoids [96]. In addition, some evidence indicated that K252a also inhibited growth [82,92], abolished invasion [98] and reduced metastasis [82,95] of breast cancer. However, as mentioned above, Tagliabue et al. [100] found that TrkA cooperates with HER2 to activate breast cancer cell proliferation under NGF stimulation. Indeed, the TrkA phosphorylation inhibitor K252a did not affect the NGF-mediated activation of HER2, suggesting also targeting these receptors for the inhibition of breast cancer cell proliferation. Moreover, the use of other TrkA inhibitors, such as LY294002 and PD98059, led to a complete abolition of NGF-induced invasion in the MDA-MB-231 cell line [98] whereas GW441756, another TrkA inhibitor that blocks the formation of the TrkA/β1-integrin/FAK/Src complex, could reverse proliferation, migration and invasion in MDAMB-231 and MDA-MB-453 cells [91]. Trouvilliez et al. [101] tested the effects of the administration of the CD44v3 mimetic peptide 4 on the formation of the TrkA/CD44v3 complex. They found that this complex could impair clonogenicity and the invasion of breast cancer cells in vitro and tumor growth and metastasis in vivo [101]. Moreover, Zhang et al. [103] demonstrated that the downregulation of TrkA receptors through the siRNA in MCF-7 cell and tumor xenograft mice model inhibited the proliferation of cancer cells and arrested the cell cycle at G0/G1 phase via inactivation of NF-κBp65. Moreover, TrkA siRNA also increased the efficacy paclitaxel and decreased the presence of lung metastasis in tumor xenografted mice. Moreover, Demont et al. [18] reported that the pharmacological inhibition of TrkA with K252a and siRNA and of sortilin with siRNA resulted in the abolition of proNGF-induced invasion and migration in different breast cancer cell lines [18]. Lévêque et al. [19] also found that the simultaneous inhibition of TrkA via lestaurtinib and siRNA and of EphA2 via siRNA reduced the breast tumor aggressiveness.

In addition to NGF and TrkA, other researchers targeted NGFR/p75NRT signaling pathways for the treatment of breast cancer. As previously mentioned, the binding of NGF to NGFR/p75NTR leads to the activation of NF-kB or JNK, which mediate opposite effects on survival and apoptosis, respectively [10]. Descamps et al. [90] tested the use of the NF-kB pharmacological inhibitor SN50 in the MCF-7 cell line, finding a reduction of NGF antiapoptotic activity. In addition, the anti-apoptotic effect of NGF is reduced by the transfection of MCF-7 cells with IkBm. Bashir et al. [84] showed that the treatment with thymoquinone of MCF-7 and MCF7-CSCs cells downregulated mRNA expression of NGFR/p75NTR and its downstream target NF-κB. Thymoquinone also altered the expression of the target gene of the NF-κB pathway, such as Sox2 and Nanog, involved in proliferation and survival of cancer cells and cancer stem cells. Chakravarthy et al. [17] demonstrated that the knock-down of NGFR/p75NTR using short hairpin RNA (shRNA) or small molecule inhibition of NGF-NGFR/p75NTR interaction (i.e., Ro 08-2750) sensitized TNBC cells to the apoptosis induced by the cytotoxic/genotoxic drugs used as adjuvant therapies in breast cancer treatment. As previously reported, Wu et al. [15] showed that NGFR/p75NTR exerted its premetastatic effects by binding with TrkC, primarily through a ligand-independent manner in TNBC cells. Interestingly, the use of shNGFR/p75NTR and shTrkC to silence the gene expression of the two receptors reduced invasive capacity in vivo and sphere growth *in vitro*, respectively, and increased the sensitivity of TNBC cells to the anti-Trk drug entrectinib. As mentioned above, Naderi et al. [97] identified a novel subtype of ER-positive breast cancer, characterized by the overexpression of the *BEX2* gene, which regulates the NGF-mediated inhibition of apoptosis through NF-kB activation. Notably, the authors also found that, in response to estradiol and tamoxifen, *BEX2* modulates the apoptosis of breast cancer cells. Furthermore, *BEX2* overexpression enhances the antiproliferative effect of tamoxifen, suggesting the involvement of the NGF/BEX2/NF-kB pathway in the modulation of the response to the hormonal drug in primary breast cancer.

## 6. Conclusions

**Figure 2:**
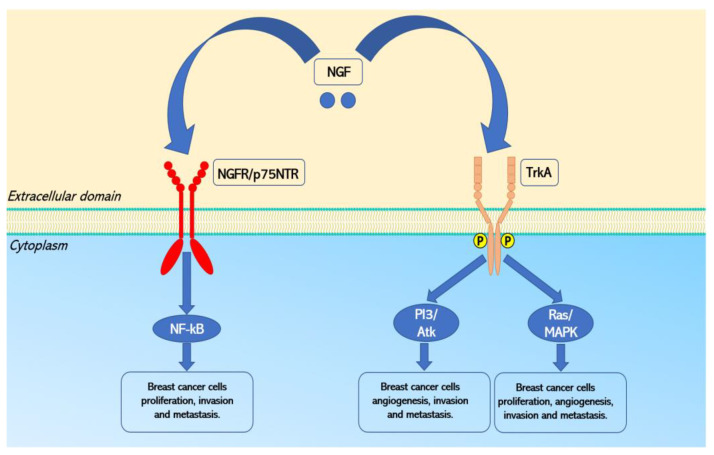
Signaling pathways activated by NGF in breast cancer. Note. NF-κB, Nuclear factor-κB; PI3K, Phosphatidylinositol-3-kinase; Atk, serine-threonine kinase; Ras, Rat sarcoma virus proteins; MAPK, Mitogen activated protein kinase.

In conclusion, the evidence reported and discussed in this systematic review demonstrated the pivotal role of pro-NGF, NGF and their receptors’ expression in breast cancer development, proliferation, growth, angiogenesis, migration, invasion and metastasis (Figure 2). These characteristics make pro-NGF, NGF, TrkA and NGFR/p75NTR good candidates as diagnostic and prognostic tools and therapeutic targets for different types of breast cancer, as indicated by other lines of research.

## Figures and Tables

**Table 1:** The main types of classification of breast cancer.

	Breast Cancer Classification		
	*General classification *	*Type*	*Sub-type*
**Histological Type**	Carcinoma in situ	Ductal	Comedo Cribiform Micropalillary Papillary Solid




		Lobular	
	Invasive or infiltrating Carcinoma	Tubular	
		Ductal lobular	
		Invasive lobular	
		Infiltrating ductal	
		Mucinous	
		Medullary	
	*Grade*	*Differentiation*	*Elston-Ellis Score*
**Grading**	Grade 1 (G1)	Well	3–5
	Grade 2 (G2)	Moderate	6–7
	Grade 3 (G3)	Poor	8–9
	*Molecular markers*		*Cases*
**Immunophenotype**	HR positive/HER2 negative		70%
	HER2 positive		15–20%
	Triple-negative		~15%
	*Stage*	TNM	Category
**TNM classification**	0	Tis N_0_ M_0_	Carcinoma in situ
	I	T_1_, N_0_, M_0_	Early breast cancer
	II	T_1_, N_1_, M_0_ T_2_, NO_1_, M_0_	Early breast cancer
	III	Any T, N_2–3_, M_0_, T_3_ Any N, M_0_	Locally advanced
	IV	Any T, any N M_1_	Metastatic
	*Subtype*		*Cases*
**Molecular subtype**	Luminal A		~40%
	Luminal B		~20%
	Basal-like		15–20%
	Claudin-low		12–14%
	HER2-enriched		10–15%

**Table 2:** Overview of included studies.

Authors	Title	Year of Publication	Type of Study	Reference
Adriaenssens et al.	Nerve Growth Factor Is a Potential Therapeutic Target in Breast Cancer	2008	ex vivo and in vivo	[82]
Aragona et al.	Nerve Growth Factor Receptor Immunoreactivity in Breast Cancer Patients	2001	ex vivo	[83]
Bashir et al.	Upregulation of CD271 transcriptome in breast cancer promotes cell survival via NFκB pathway	2022	ex vivo and in vitro	[84]
Chakravarthy et al.	Nerve growth factor (NGF)-mediated regulation of p75NTR expression contributes to chemotherapeutic resistance in triple negative breast cancer cells	2016	ex vivo and in vitro	[17]
Chiarenza et al.	Tamoxifen Inhibits Nerve Growth Factor-induced Proliferation of the Human Breast Cancerous Cell Line MCF-7	2001	in vitro	[85]
Com et al.	Nerve Growth Factor Receptor TrkA Signaling in Breast Cancer Cells Involves Ku70 to Prevent Apoptosis	2007	in vitro	[86]
Davidson et al.	Altered expression and activation of the nerve growth factor receptors TrkA and p75 provide the first evidence of tumor progression to effusion in breast carcinoma	2004	ex vivo and in vitro	[87]
Demont et al.	Pro-nerve Growth Factor Induces Autocrine Stimulation of Breast Cancer Cell Invasion through Tropomyosin-related Kinase A (TrkA) and Sortilin Protein	2012	ex vivo and in vitro	[18]
Descamps et al.	Nerve Growth Factor Is Mitogenic for Cancerous but Not Normal Human Breast Epithelial Cells	1998	in vitro	[88]
Descamps et al.	Expression of Nerve Growth Factor Receptors and Their Prognostic Value in Human Breast Cancer	2001a	ex vivo	[89]
Descamps et al.	Nerve Growth Factor Stimulates Proliferation and Survival of Human Breast Cancer Cells through Two Distinct Signaling Pathways	2001b	in vitro	[90]
Di Donato et al.	Targeting the Nerve Growth Factor Signaling Impairs the Proliferative and Migratory Phenotype of Triple-Negative Breast Cancer Cells	2021	in vitro	[91]
Dolle’ et al.	Nerve growth factor overexpression and autocrine loop in breast cancer cells	2003	ex vivo and in vitro	[92]
Islam et al.	Nerve growth factor from Indian Russell’s viper venom (RVV-NGFa) shows high affinity binding to TrkA receptor expressed in breast cancer cells: Application of fluorescence labeled RVV-NGFa in the clinical diagnosis of breast cancer	2020	ex vivo and in vitro	[14]
Jung et al.	Elevated Level of Nerve Growth Factor (NGF) in Serum-Derived Exosomes Predicts Poor Survival in Patients with Breast Cancer Undergoing Neoadjuvant Chemotherapy	2021	in vivo	[93]
Kumar et al.	Localization and hypersecretion of nerve growth factor in breast phyllodes tumors: Evidence from a preliminary study	2020	ex vivo	[94]
Kyker-Snowman et al.	TrkA overexpression in non-tumorigenic human breast cell lines confers oncogenic and metastatic properties	2020	in vitro and in vivo	[70]
Lagadec et al.	TrkA overexpression enhances growth and metastasis of breast cancer cells	2009	in vitro and in vivo	[95]
Lévêque et al.	ProNGF increases breast tumor aggressiveness through functional association of TrkA with EphA2	2019	in vitro and in vivo	[19]
Melck et al.	Suppression of Nerve Growth Factor Trk Receptors and Prolactin Receptors by Endocannabinoids Leads to Inhibition of Human Breast and Prostate Cancer Cell Proliferation	2000	in vitro	[96]
Naderi et al.	BEX2 Is Overexpressed in a Subset of Primary Breast Cancers and Mediates Nerve Growth Factor/Nuclear Factor-KB Inhibition of Apoptosis in Breast Cancer Cell Lines	2007	ex vivo and in vitro	[97]
Noh et al.	Expression of nerve growth factor and heme oxygenase-1 predict poor survival of breast carcinoma patients	2013	ex vivo	[16]
Romon et al.	Nerve growth factor promotes breast cancer angiogenesis by activating multiple pathways	2010	in vitro and in vivo	[98]
Sakamoto et al.	Combined evaluation of NGF and p75NGFR expression is a biomarker for predicting prognosis in human invasive ductal breast carcinoma	2001	ex vivo	[99]
Tagliabue et al.	Nerve Growth Factor Cooperates with p185*HER2* in Activating Growth of Human Breast Carcinoma Cells	2000	ex vivo and in vitro	[100]
Trouvilliez et al.	Direct interaction of TrkA/CD44v3 is essential for NGF-promoted aggressiveness of breast cancer cells	2022	in vitro and in vivo	[101]
Tsang et al.	Nerve growth factor receptor (NGFR): a potential marker for specific molecular subtypes of breast cancer	2013	ex vivo	[102]
Wu et al.	Nerve growth factor receptor increases the tumor growth and metastatic potential of triple-negative breast cancer cells	2021	in vitro and in vivo	[15]
Zhang et al.	Blockage of tropomyosin receptor kinase a (TrkA) enhances chemo-sensitivity in breast cancer cells and inhibits metastasis *in vivo*	2015	in vitro and in vivo	[103]
